# Fully digital versus conventional workflow: Are removable complete overdentures equally good? A randomized crossover trial

**DOI:** 10.1111/cid.13398

**Published:** 2024-09-30

**Authors:** Thomas Van de Winkel, Frans Delfos, Olleke van der Heijden, Ewald Bronkhorst, Luc Verhamme, Gert Meijer

**Affiliations:** ^1^ Department of Oral Maxillofacial Surgery Radboud University Medical Center Nijmegen The Netherlands; ^2^ Dental Laboratory, Department of Dentistry Radboud University Medical Center Nijmegen The Netherlands; ^3^ Samenwerkende Tandartsen, Colosseum Dental Nijmegen The Netherlands; ^4^ Radboud Institute of Health Sciences, Department of Dentistry Radboud University Medical Center Nijmegen The Netherlands

**Keywords:** CAD/CAM, digital workflow, edentulous, implant overdenture, OHIP‐20, OHRQoL, PROMs

## Abstract

**Introduction:**

Implant‐supported removable complete overdentures (IODs) are a common treatment in case of edentulism and malfunctioning of the conventional denture. Manufacturing IODs in a conventional way (C‐IODs) is time‐consuming, but in a digital workflow, this can be done in three sessions. Digitally produced IODs (3D‐IODs) are also more advantageous than C‐IODs because lost or broken 3D‐IODs can be swiftly reproduced as the digital design is always available.

**Purpose:**

To prove in a non‐inferiority study, with a margin of 0.3 point per Oral Health Impact Profile‐20 (OHIP‐20) question, that IODs made according to a fully digital workflow (3D‐IODs), function as good as C‐IODs with respect to patient‐reported outcome measures (PROMs).

**Materials and Methods:**

This randomized crossover study included 36 fully edentulous patients who showed extreme resorption of the maxillary alveolar process, making denture retention difficult. After a maxillary bone augmentation and the installation of 4–6 implants, each patient wore both types of IOD for 1 year each, with the order reversed in two subsets of patients. The 3D‐IODs and C‐IODs were fabricated in advance for both jaws (at least two mandibular implants were already present).

The OHIP‐20 survey was performed at baseline, after 1 year (before the IOD switch), and after 2 years to determine patient satisfaction scores using a visual analog scale (VAS). The general health status was assessed using the Short Form (SF‐36) questionnaire.

**Results:**

Regarding the PROMs, patients preferred the 3D‐IOD: the improvement on the overall OHIP scale (0–4), expressed as a mean, was 0.26 points greater than for the C‐IOD (*p* < 0.001). This applied also to the VAS scale (1–100) with an increase of 7.37 points (*p* < 0.001). Regarding the SF‐36 scale, only for the item “emotional well‐being,” the 3D‐IOD scored significantly better (*p* = 0.033).

**Conclusion:**

Compared with conventionally fabricated C‐IODs, fully digitally produced 3D‐IODs resulted in significantly higher OHIP‐20 and satisfaction scores.

**Summary Box**

**What is known**

- Implant‐supported removable complete overdentures (IODs) are a commonly accepted treatment option in case of edentulism. The design and manufacturing of a conventional IOD (C‐IOD) is time‐consuming for both the patient and healthcare provider and can be shortened using a digital workflow. The OHIP‐20 is a renowned questionnaire for measuring oral health‐related quality of life in edentulous patients.

**What this study adds**

- After wearing both the C‐IOD and the digitally designed and manufactured IOD (3D‐IOD) for 1 year each in a randomly assigned order, patient‐reported outcome measures were significantly better for the 3D‐IOD.

## 1 INTRODUCTION

Since 2001, edentulism has been classified as a physical disability by the World Health Organization^1^ as it leads to chewing difficulties and compromised aesthetics. In the United States alone, it is estimated that approximately one‐third of adults aged ≥65 years are edentulous.^2^ Although a decline in edentulism rate has been reported in the United States and other Western countries, this drop is more than compensated by the growth of the adult population over 55 years of age.^3^ Edentulism thus remains an important clinical topic.

The first step in the treatment of edentulism is the placement of a complete denture (CD). Unfortunately, CDs only partially restore aesthetics and chewing capacity.^4^ An additional drawback is the CD‐induced compressive force during chewing, leading to an ongoing process of jawbone resorption, complicating the retention and stability of the CD. Consequently, denture rubbing causes pain, which makes daily life more difficult, both in a physical and psychological sense.^5^

To overcome CD‐related complaints, an implant‐supported overdentures (IOD) retained by two interforaminal implants is considered the first choice of treatment for the edentulous lower jaw.^6^ Studies on mandibular IODs typically report a successful outcome, as they result in a high patient satisfaction (PS), upgrade the oral health‐related quality of life (OHRQoL), and improve the chewing capacity.^6^, ^7^, ^8^, ^9^, ^10^, ^11^, ^12^ When used in the upper jaw, implant treatment also enhances stability, function, speech, and PS.^13^, ^14^, ^15^, ^16^, ^17^ In addition, implants prevent progressive alveolar bone loss.^18^ Disadvantages include the invasive nature, the need for maintenance, high costs, and the risks of peri‐implantitis.

After osseointegration of the implants, an IOD or an implant‐fixed prosthesis (IFP) can be installed. Of these two options, IFPs initially seem to be the best solution; however, an IOD may be the better choice in cases of reduced posterior bone quality, anatomical limitations, and systemic medical conditions.^19^, ^20^, ^21^ Furthermore, IODs are documented to be less invasive and more economical than IFPs.^22^, ^23^, ^24^ In the event of prosthetic complications, repairs can be executed more easily for removable IODs than IFPs. If the IOD is manufactured using a digital workflow, prostheses can be reproduced more quickly when lost or broken, because the digital design always remains available.^25^

The high costs of IFPs have led Dutch national insurance companies to reimburse a maximum of six implants in the edentulous maxilla and two in the edentulous mandible on the condition that (1) an extremely resorbed alveolar process is present, and (2) only IODs, so no fixed prostheses, are delivered.^26^

IODs can be manufactured in a conventional way (C‐IODs) or with the use of a fully digital workflow (3D‐IOD).^27^ Data were gathered concerning patient‐reported outcome measures (PROMs) for both C‐IODs and 3D‐IODs. These data were collected at baseline, and after 12 and 24 months of treatment. With respect to the PROMs, it was hypothesized that patients would rate both C‐IOD and 3D‐IOD as equivalent.

## 2 MATERIALS AND METHODS

The present study was conducted in accordance with the Declaration of Helsinki and approved by the Ethics Committee of Arnhem/Nijmegen, NL 2017‐3671, December 12, 2017 (Dossier number: 2017‐3671 NL‐number: NL63073.091.17). The article was drafted according to the “Checklist for Non‐inferiority and Equivalence Trials,” which conform the CONSORT guidelines.^28^ The raw data are archived in the “Data Archiving and Networked Services” (DANS) of the Royal Netherlands Academy of Arts and Sciences (KNAW) under the Persistent identifier 10.17026/dans‐25s‐6cdk and hence are accessible for the public.

For the PROMs, the OHRQoL was measured using the organ‐specific Oral Health Impact Profile‐20 (OHIP‐20) questionnaire. PS was scored using a visual analog scale (VAS). To inventory the IOD impact on general well‐being and functioning, the Short Form 36 Questionnaire (SF‐36) instrument was completed, as suggested by Heydecke et al.^13^

This non‐inferiority study entailed a randomized crossover prospective clinical trial. With respect to OHIP‐20 and PS‐VAS scores, it was hypothesized that patients would rate both the C‐IOD and 3D‐IOD as similarly beneficial.

### 2.1 Trial design

Patients were asked to wear the C‐IOD or 3D‐IOD for 1 year before alternating to the other type for a second year. At baseline, and after each year of wearing one of the IOD types, the patients responded to the following PROMs: the OHIP‐20 questionnaire,^29^ the PS‐VAS, and the SF36 health questionnaire.^30^

From the primary outcomes, the biggest differences were expected for the OHIP‐20; therefore, a power calculation was focused precisely on this. The sample size calculation was performed using the available literature data, with the aim of obtaining about the same number of OHIP points for the 3D‐IOD and C‐IOD after 1 year of use. This crossover randomized prospective study was analyzed as a non‐inferiority study, with a power of an 80% chance of detecting 3D‐IODs scoring six points less (inferiority margin) than the C‐IODs. It was not feasible to calculate the required sample size based on a multilevel model; therefore, the sample size was determined using a t‐test approach, which can be expected to have a similar power. The standard deviation (sd) of the OHIP‐49 for comparable populations is approximately 31,^31^ which was translated into a sd of 13 for the OHIP‐20. As within‐person comparisons were performed, the correlation between two measurements in one person was estimated to be 0.5, meaning a gain of a factor 1/√2 in the sd as compared with a parallel group design. Using an alpha of 0.05, a power of 80%, an inferiority margin of 6, and a sd of 13/√2 = 9.2, this implied that our randomized prospective study must include 30 subjects, wearing both a C‐IOD and a 3D‐IOD. In addition, as two treatment centers were to be included in this crossover trial, a fixed effect for center had to be included in the analysis, slightly reducing the power. Combining this with the loss to withdrawal of patients, estimated to be six at most, a total of 36 patients needed to be included.

Participants were randomly assigned to either the A group, which started with a 3D‐IOD and subsequently wore the C‐IOD, or to the B group, which started wearing the C‐IOD and ended with the 3D‐IOD. For an optimal comparison, it was decided that equal groups should be formed; 18 patients were assigned to the A group and 18 to the B group.

Assignment to group A or B was executed with a 1:1 allocation as per a computer‐generated randomization schedule and using permuted blocks of random sizes. The block sizes were not disclosed. This procedure enabled the evaluation of preliminary data, which facilitated the optimization of both the treatments and procedures during the study. The participants were randomized using an online randomization tool.

An independent secretary prepared a stack of sealed study envelopes noting the group (A or B), which was opened in sequence only by the clinician treating the patients. After the patient had given permission to participate in the study, the secretary announced to the treating dentist which type of IOD should be placed. The order of patient registration determined which envelope had to be opened. As such, both patient and investigator were blinded.

### 2.2 Centers

Two centers were involved: a dental clinic affiliated with the Radboud University Medical Center (Radboudumc) in Nijmegen, the Netherlands, and a center for special dental care at the Amphia Hospital in Breda, the Netherlands. In each center, a lockable filing cabinet was present, in which the individual case report forms (CRF) containing the clinical scores and all questionnaires were stored.

### 2.3 Study population

All included patients were edentulous in both the upper and lower jaw and suffered from eating deficiency, loosening of their CDs during speaking, and feelings of embarrassment about their appearance. Cone beam computer tomography (CBCT) imaging confirmed the assumption of an extreme loss of alveolar bone in the maxilla. To make implant installation feasible, all participants needed an extensive maxillary bone augmentation procedure. Permission was obtained from the health insurance company for all patients prior to treatment, which covered all costs except for a relatively small personal contribution. If no implants were present in the lower jaw, mandibular implants were also placed there during the installation of the maxillary implants.

Patients with uncontrolled systemic diseases, an immune‐compromised status, those who had been previously treated with oral or intravenous bisphosphonates, or who had undergone prior radiotherapy in the maxillofacial area were excluded from the study, as well as smokers. Patients were urged to remove their IODs at night, thus preventing the overloading of the implants.

Ethnic backgrounds are not reported in the literature surrounding IOD satisfaction. With respect to age and gender, no differences in satisfaction were observed for the IODs^32^, ^33^; therefore, no distinction was made for these items when reporting patient results.

Because the 3D‐IOD was made during the same sessions as the C‐IOD, the patients were not informed whether they would start with the 3D‐IOD (group A) or with the C‐IOD (group B) type first. Furthermore, researchers and research assistants were blinded when assessing the data outcome and statistical analyses. To anonymize the data, the patients were coded using the first letter of the research location, B(reda) or N(ijmegen), followed by the serial number of inclusions. All research data were imported into Castor™ (New York, USA).

### 2.4 Surgery

In Nijmegen, all 29 patients underwent a bone augmentation procedure. Cortico‐cancellous bone blocks were harvested under general anesthesia from the superior anterior medial iliac crest.^34^ After performing a sinus floor elevation procedure, vertical augmentation was achieved by applying bone chips.^35^, ^36^ To broaden the upper jaw, bone blocks were fixated against the alveolar process with the use of osteosynthesis screws (2.0‐mm Champy System, KLS Martin, Tüttingen, Germany). Effort was made to position the screws in the most horizontal position^37^ to allow optimal support for the guided template in a later stage.

In Breda, three patients were treated as described above, while the other four patients only underwent a sinus floor elevation procedure.

### 2.5 Implants

In Nijmegen, a total of 4–6 maxillary Nobel Parallel Conical Connection™ implants (Nobel Biocare, Kloten, Switzerland) were planned for each patient based on CBCT images using the Procera Clinical Design™ software (Nobel Biocare). Parallelism of the implants was pursued, and care was taken to position at least the apex of the implants into the original maxillary bone. Furthermore, peri‐implant bone thickness was aimed to be at least 2 mm.^38^ Hereafter, a surgical template was printed to install the implants according to the NobelGuide™ procedure (Nobel Biocare), which means that the implants underwent flapless placement. To create optimal template stability, the osteosynthesis screws were partially unscrewed before the implant installation to allow the template to rest on them.^37^ If not already present, two Nobel Parallel Conical Connection™ implants were installed in the symphysis region of the lower jaw.

In Breda, all patients received 4–6 Straumann Tissue Level Implants (Standard Plus) with a diameter of 4.1 mm (Institute Straumann, Basel, Switzerland) in their upper jaw. An open procedure was executed, beginning with the preparation of a subperiosteal flap. Implants had already been installed in the lower jaws of these patients.

In all augmented patients, the maxillary bone volume was sufficient at the time of implantation. Healing abutments were positioned onto all implants. Two weeks later, patients were allowed to wear their dentures again; sufficient space was created around the abutments and a fresh relining was conducted. The use of denture adhesive was permitted. The relined upper denture was checked every 6 weeks for any overloading of the abutments.

### 2.6 Prosthetics

**FIGURE 1:**
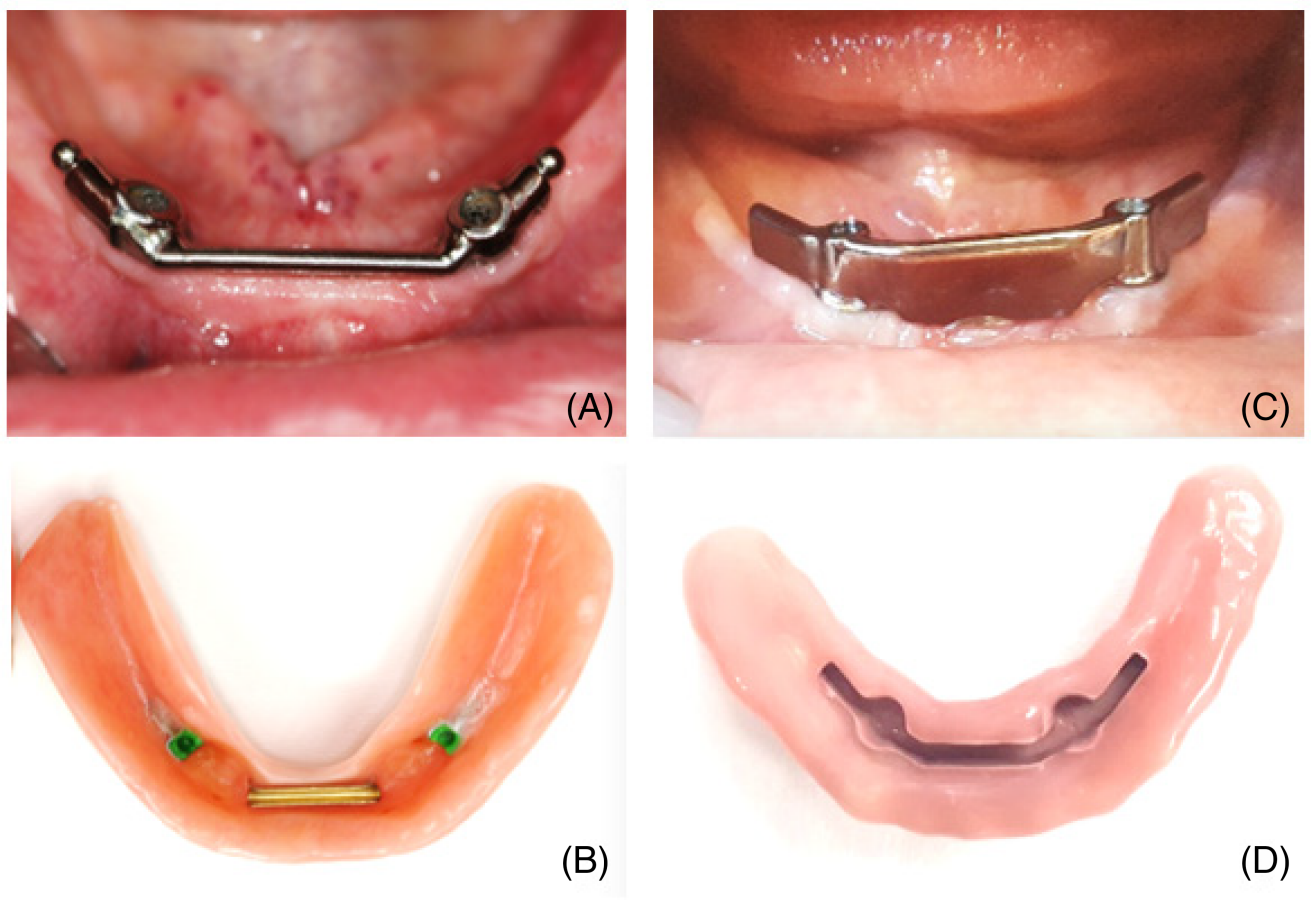
The mandible: (A) Intraoral view of a conventional bar including ball attachments, (B) Inside the C‐IOD the green VKS matrices are visible, (C) Bar suitable for the PEEK sliding attachment, (D) Inside the 3D‐IOD.

**FIGURE 2:**
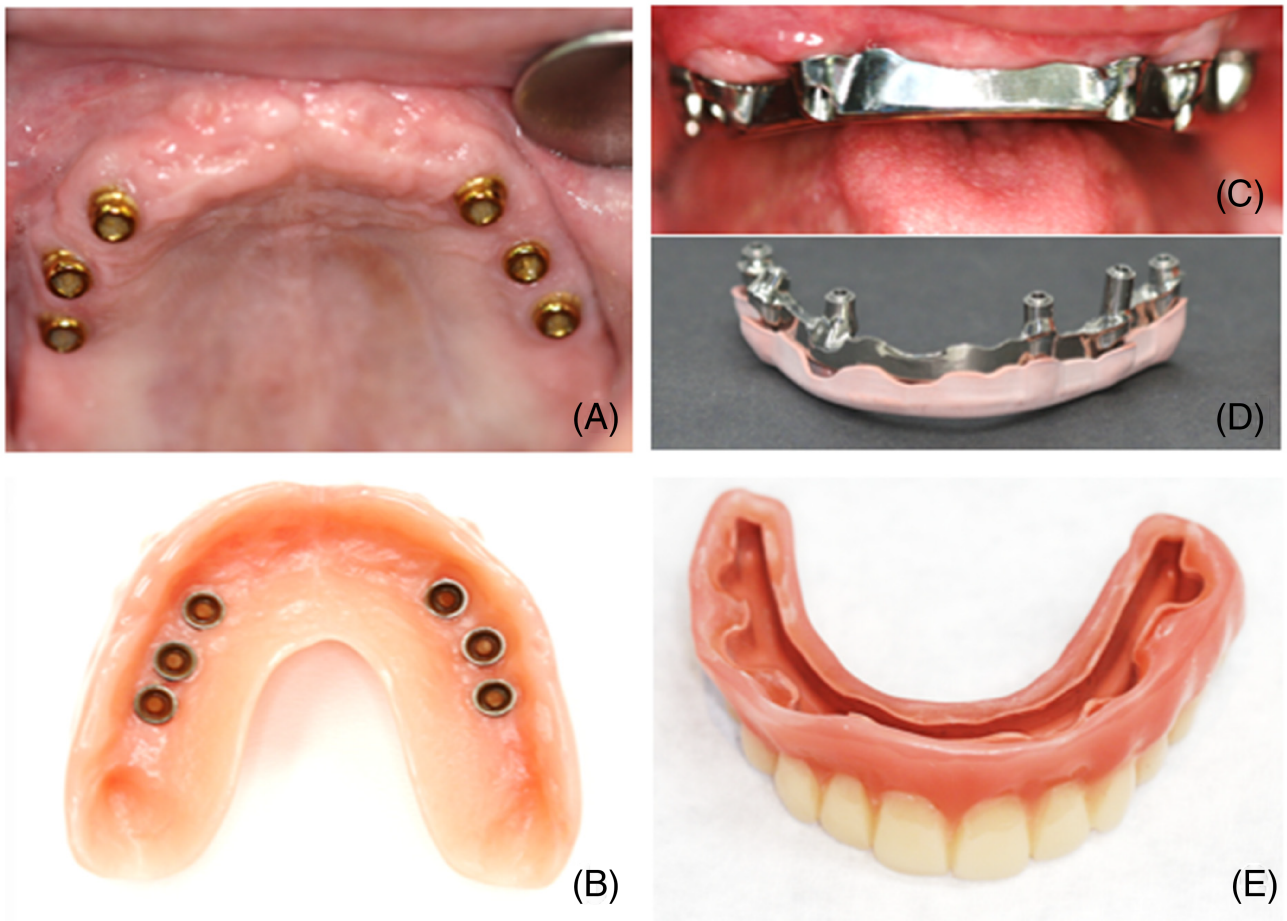
The maxilla: (A) Intra‐oral view of the maxillary Locators. (B) Inside the upper C‐IOD, showing the nylon rings. (C) 3D bar suitable for the PEEK sliding attachment. (D) The thin (2‐mm) 3D bar in combination with the PEEK attachment. (E) Inside the 3D‐IOD, into which the PEEK is glued.

In advance of the study, a final C‐IOD and 3D‐IOD were fabricated for each patient (Figures 1 and 2). The treatment sessions were combined to hide from the patient which techniques were used for which IOD type. The identity of the installed IOD remained hidden from both the patients and researcher (TW). After opening the research envelope, only the clinician knew which IOD type was to be installed first.

### 2.7 C‐IOD

C‐IODs were manufactured in 5–6 clinical sessions. First, a preliminary impression of the patient's mouth was taken using stock trays. After pouring the impression in dental stone, custom trays were fabricated. At the second appointment, final impressions were taken, representing both the soft tissue and the exact position of the implants. In the third session, the centric occlusion, vertical dimension of occlusion, and position of the teeth were registered.

In Nijmegen, a milled titanium bar with distal extensions (Atlantis™, Charlotte, North Carolina, USA) together with a Dolder matrix (Cendres Metaux, Biel, Switzerland) was used as a click‐retention system for the lower implants (Table 1). To provide additional retention to the C‐IOD, the mandibular titanium bar ended in a ball attachment combined with a VKS™ matrix (Bredent Medical, Senden, Germany; Figure 1A,B). For the upper implants, the treating dentist preferred the Locator™ system (ZEST Anchors, Carlsbad, California, USA) (Figure 2A,B). In Breda, a milled titanium bar (Atlantis™) together with a Dolder matrix (Cendres Metaux) was used for implants in both the upper and lower jaws (Table 1).

**TABLE 1:** Number of patients and type of click‐attachment: per center, for both maxilla and mandible.

Center	Number of patients	Maxilla: C‐IOD on Locators™ versus 3D‐IOD on 3D‐bar	Maxilla: C‐IOD on bar versus 3D‐IOD on 3D‐bar	Mandible: C‐IOD on bar versus 3D‐IOD on 3D‐bar	Mandible: Solely C‐IOD on bar (thus no 3D‐IOD)
Nijmegen	27	27		18	9
Breda	5		5	4	1
Total	32	27	5	22	10

A “wax‐up” was then made, in which commercially available acrylic teeth were arranged. At the fourth visit, the “C‐IOD in wax” was checked for fit, function, and aesthetics, together with the click‐retention system. Changes to the design could still be made at this stage. If this was the case, an additional clinical session was indicated. As a final step, the C‐IOD was heat‐pressed using two‐component polymethylmethacrylate (PMMA) in the dental laboratory and placed.

### 2.8 3D‐IOD

The creation of a 3D‐IOD using a fully digital workflow has been extensively described.^27^ In the first clinical session, five steps need to be followed. The 3Shape Smile Design™ (3Shape, Copenhagen, Denmark) program was used to identify the patient's expectations (step 1). After loading the patient's facial image, a preliminary digital setup was projected onto it. The aesthetic items discussed included the shape and color of the projected teeth, as well as their visibility (step 2).

Subsequently, digital data were collected to create a virtual head. The existing dentures were checked to determine whether they were still in centric occlusion (step 3). After scanning (TRIOS 3™; 3Shape) both the upper and lower dentures (step 4), an intra‐oral scan of the maxilla and mandible was taken with scan bodies (TRIOS 3™) mounted onto the implants (step 5). Matching each individual scanned CD with a scan of both CDs in occlusion allowed the correct maxilla‐mandibular relationship to be captured.

In the first laboratory session, after importing all data into the TRIOS Design Studio program (3Shape™), the final 3D‐IOD was designed. Digitally, the shape, size, and position of each individual tooth could still be adjusted. In the Real View™ tool, all modifications were immediately exhibited in the facial photograph. Furthermore, the titanium bar was digitally engineered down to the implant level (Figures 1C,D and 2C–E), with a frictional guide plane to provide excellent retention and stabilization of the 3D‐IOD.^39^ In the same session, the matrix, that is, the future sliding mechanism made of polyetheretherketone (PEEK), was digitally designed (Figure 2D,E). The bars were milled from Core Titanium™ blocs (Core3dcentres, Las Vegas, Nevada, USA), and the matrices from PEEK discs (Dental Direkt™, Spenge, Germany). All components were assembled and secured in a printed trial IOD.

In the second clinical session, the fit of the bar, vertical dimension of occlusion, centric occlusion, tooth alignment, tooth size, and lip support were checked in the patient. If necessary, changes were made to the trial IOD, which was then rescanned.

In the second laboratory session, after all adjustments were digitally imported into the TRIOS Design Studio program (3Shape™), the final IOD was manufactured. Using computer‐aided design and manufacture (CAD‐CAM), the final teeth were milled from a DDpolyXML disc (Dental Direkt™), which consists of a multilayer acrylic resin polymer. The IOD base itself was milled from a block VITA Vionic Base™ (VITA Zahnfabrik, Bad Säckingen, Germany). The teeth were then bonded to the base using Vita Vionic™ Bond (VITA Zahnfabrik, Bad Säckingen, Germany). Compared with heat polymerized acrylic resin and 3D printed resins, CAD‐CAM milled resins exhibit greater flexural properties and hardness.^40^

In the third clinical session, both titanium bars were placed onto the implants and the mandibular and maxillary 3D‐IOD were installed.

### 2.9 PROMs

#### 2.9.1 Oral health impact (OHIP‐20)

To assess the OHRQoL, patients were asked to complete the OHIP‐20 questionnaire at baseline, after 12 months at the exchange from IOD type A to B (or vice versa), and after 24 months at the end of the study. The OHIP‐20 is recommended for use in edentulous patients. Earlier findings show that the OHIP‐20 possesses good psychometric properties of value for clinical trials, particularly for oral prostheses.^29^

The OHIP‐20 comprises 20 questions covering seven domains: functional limitation, pain, psychological discomfort, physical disability, psychological disability, social disability, and handicap. The response to each question was rated on a Likert scale ranging from 0 for “never” to 4 for “very often”, meaning the total OHIP‐20 score ranges from 0 to 80, with a lower score indicating a higher OHRQoL.

Data from the OHIP‐20 were presented as a sum score, that is, the score of all 20 questions added together. However, to facilitate the comparisons of analyses between both the overall OHIP as well as its individual domains, it was decided to present the improvement on the overall OHIP scale (0–4), expressed as a mean.

#### 2.9.2 Patient satisfaction

At baseline and after 1 year of wearing each IOD type, the PS was rated using a VAS ranging from 1 to 100.

#### 2.9.3 SF‐36 questionnaire (QoL)

The SF‐36 questionnaire is a well‐documented generic tool that aims to assess the general health status of the population, as well as the impact of clinical and social interventions.^30^ This survey consists of eight scales yielding data on both physical and mental health (i.e., Physical functioning, Limited due to physical health, Limited due to emotional problems, Energy, Emotional well‐being, social functioning, Pain, and General health). The higher the score (0–100), the better the health.

### 2.10 Statistics

For both the OHIP‐20, as well as for PS and SF‐36 data, multilevel regression analyses (MRAs) were performed with the OHIP (or PS, or SF‐36 scale) as the dependent variable, while both experimental conditions, center, and period (year 1 vs. year 2) were considered independent variables. The variable “patient” was added as a random intercept. An analysis of variance (ANOVA) was used for the statistical test, implying that the dependent variable was not the change in OHIP‐20 (or PS, or SF‐36 scale), but instead, its value measured at the baseline, centered around its population mean, which was added to the regression model.^41^

## 3 RESULTS

### 3.1 Patients

**FIGURE 3:**
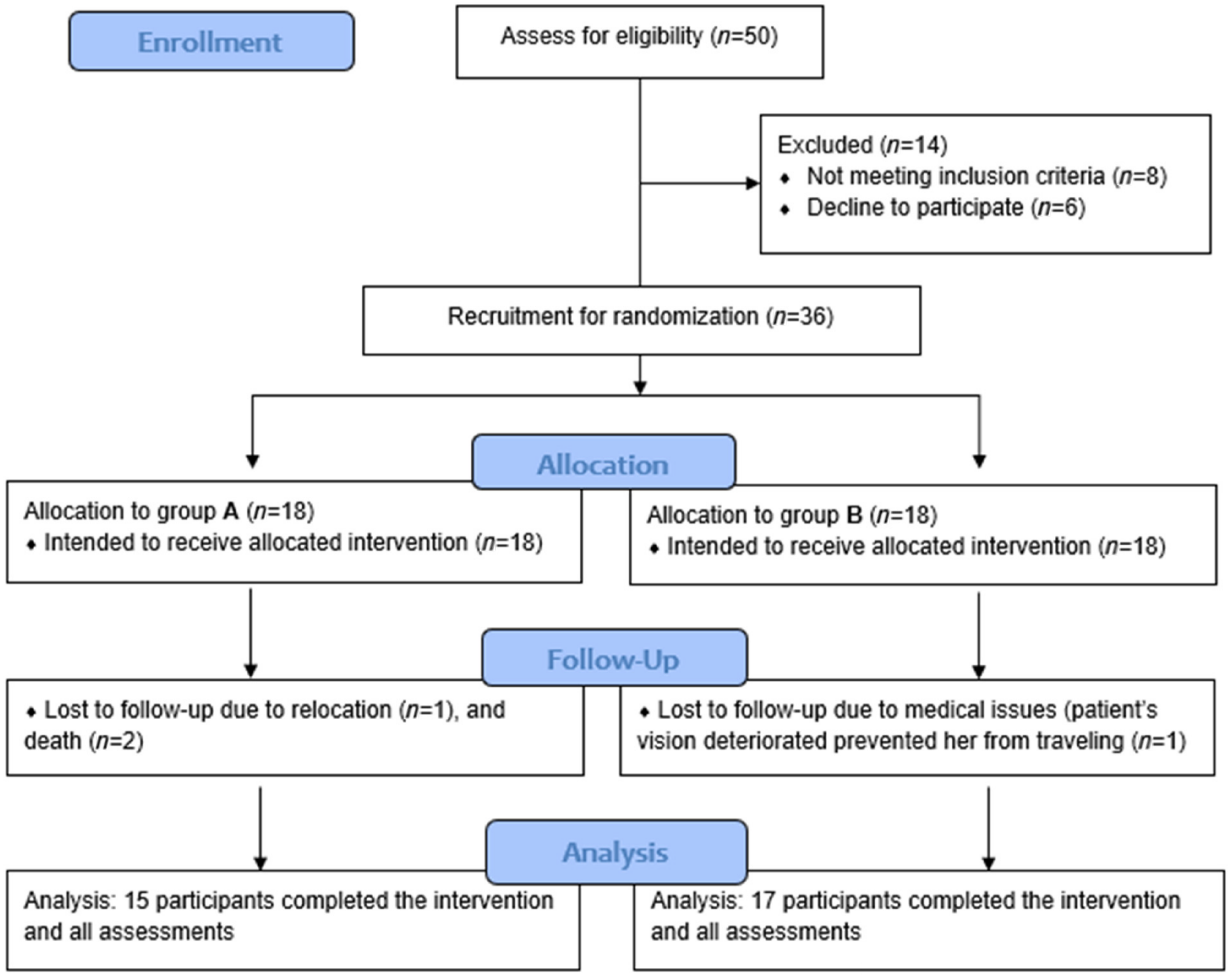
Enrollment for the study.

In total, 36 patients were included in this study (Figure 3), of whom 29 in Nijmegen (N1–N29) and seven in Breda (B1–B7). The patients were recruited between January 1, 2018, and October 1, 2019, and the study ended on December 1, 2021. Four patients discontinued the study: two had passed away (one from a heart attack, the other due to tumor metastasis); one patient's vision deteriorated, which prevented her from traveling; and another participant moved abroad. In total, 32 patients, 16 men and 16 women, completed the study. At baseline, their mean age was 62 years (sd: 6), with a range of 51–72 years.

### 3.2 Implants

A total of 181 maxillary implants were placed in 32 patients. During osteointegration, four implants failed, two of which had been placed in one patient. Another implant was lost during the first year while wearing a C‐IOD on Locators™. All five losses involved Nobel Parallel Conical Connection™ implants, representing a failure rate of 2.8% (survival 97.2%) after 2 years of function.

In the lower jaw, most patients (*n* = 30) had two implants. Each of the other two patients had four preexisting mandibular implants. None of these installed or preexisting mandibular implants (*n* = 68) failed over the course of the study.

### 3.3 Prosthetics

All 32 patients received a 3D‐IOD in their maxilla, of whom 22 also received one in their mandible (Table 1). In the remaining 10 patients, a lower C‐IOD was worn throughout the entire 2‐year study period, as relatively unknown implant brands had already been installed. These implant brands were not represented in the TRIOS™ Design Studio program (3Shape), making the creation of a 3D design impossible (Table 1).

For the upper jaw, the C‐IOD on Locators™ showed 47 complications, such as pressure ulcers, changing nylon rings, or losing housing matrices. The bar‐retained 3D‐IOD exposed 16 complications, such as pressure ulcers and correction of occlusion. For the lower jaw, the C‐IOD scored 49 complications and the 3D‐IOD a total of 39. With respect to the C‐IOD, mainly pressure ulcers and loosening of the ball attachment were observed. Regarding the 3D‐IOD mostly loosening of fixation screws of the bar was noticed.

### 3.4 Non‐inferiority analysis

**FIGURE 4:**
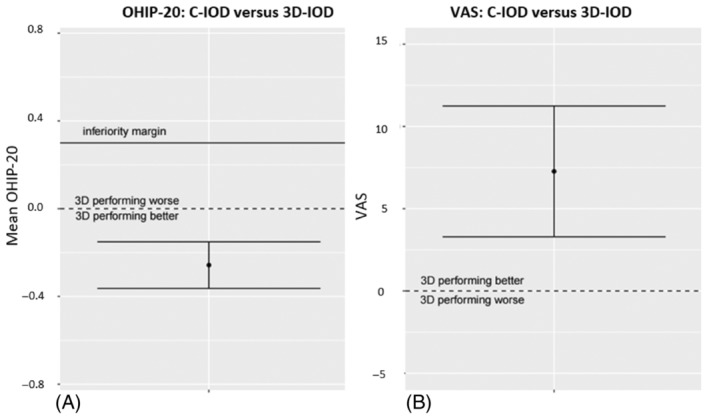
For both the OHIP‐20 (A) and patient satisfaction (VAS: 1–100) (B) the superiority analysis is depicted: Both the inferiority margin and turning point of performing “better or worse” are shown.

For the first primary outcome, the OHIP‐20 score, the non‐inferiority margin was 6 points for all OHIP‐20 questions, which equates to 0.30 per mean OHIP question. For the 3D‐IOD, the per‐OHIP question score was 0.257 lower, thus better than that of the C‐IOD (*p* < 0.001) (Figure 4A).

Also, for the VAS score, the superiority analysis showed a significantly better result (7.3‐point improvement) in the 3D‐IOD compared with the C‐IOD (Figure 4B).

## 4 PROMs

### 4.1 Oral health impact (OHIP‐20)

For the preoperative CD, the total OHIP score was 45.8. This improved to 12.0 for the C‐IOD and even further to 6.8 for the 3D‐IOD.

To facilitate the comparison between the OHIP scores, the improvement on the overall OHIP scale (0–4) was presented, expressed as a mean (Table 2).

**TABLE 2:** Main analysis of OHIP‐20 and VAS‐score: “C‐IOD versus 3D‐IOD”; first versus second period of the study; OHIP‐20/VAS scores at baseline.

Mean OHIP points					
		Estimate	*p*‐value	2.5%	97.5%
(Intercept)		1.501	<0.001*	1.184	1.818
IOD‐type	3D vs. C	−0.257	<0.001*	−0.364	−0.151
Period	2nd vs. 1st	−0.184	<0.002*	−0.290	−0.078
OHIP scores at baseline		0.171	0.069	−0.003	0.345
Center	Nijmegen vs. Breda	0.234	0.191	−0.103	0.571

VAS score					
		Estimate	*p*‐value	2.5%	97.5%
(Intercept)		76.734	<0.001*	6.928	84.186
IOD‐type	3D vs. C	7.265	<0.001*	0.329	11.238
Period	2nd vs. 1st	8.267	<0.001*	4.291	12.238
VAS scores at baseline		−0.022	0.983	−1.971	1.926
Center	Nijmegen vs. Breda	−0.9	0.823	−8.517	6.706

*Note*: OHIP scores are presented as mean OHIP‐20 points (0–4).

* *p* ≤ 0.05.

With respect to the effect of “IOD type” (Table 2), a mean improvement of 0.257 OHIP‐20 points was observed for the 3D‐IOD (*p* < 0.001).

For “period,” a significant difference in OHIP score was observed (Table 2). In the second period of the study, patients scored 0.184 points lower (better) than in the first period regardless of the type of IOD worn at that time (*p* = 0.002).

Regarding the assessment of the original CD (see “OHIP score at baseline” in Table 2), each OHIP point of disapproval at baseline also implied a more negative judgment of the final IOD irrespective of the IOD type, although this was not statistically significant (mean effect: 0.171; *p* = 0.071).

Patients treated in Breda (see “center” in Table 2) scored 0.234 points better than the patients treated in Nijmegen, although not to a significant level (*p* = 0.191). The statistical insignificance was caused by the high statistical spread (95% confidence interval [CI]: −0.103 to 0.571).

**FIGURE 5:**
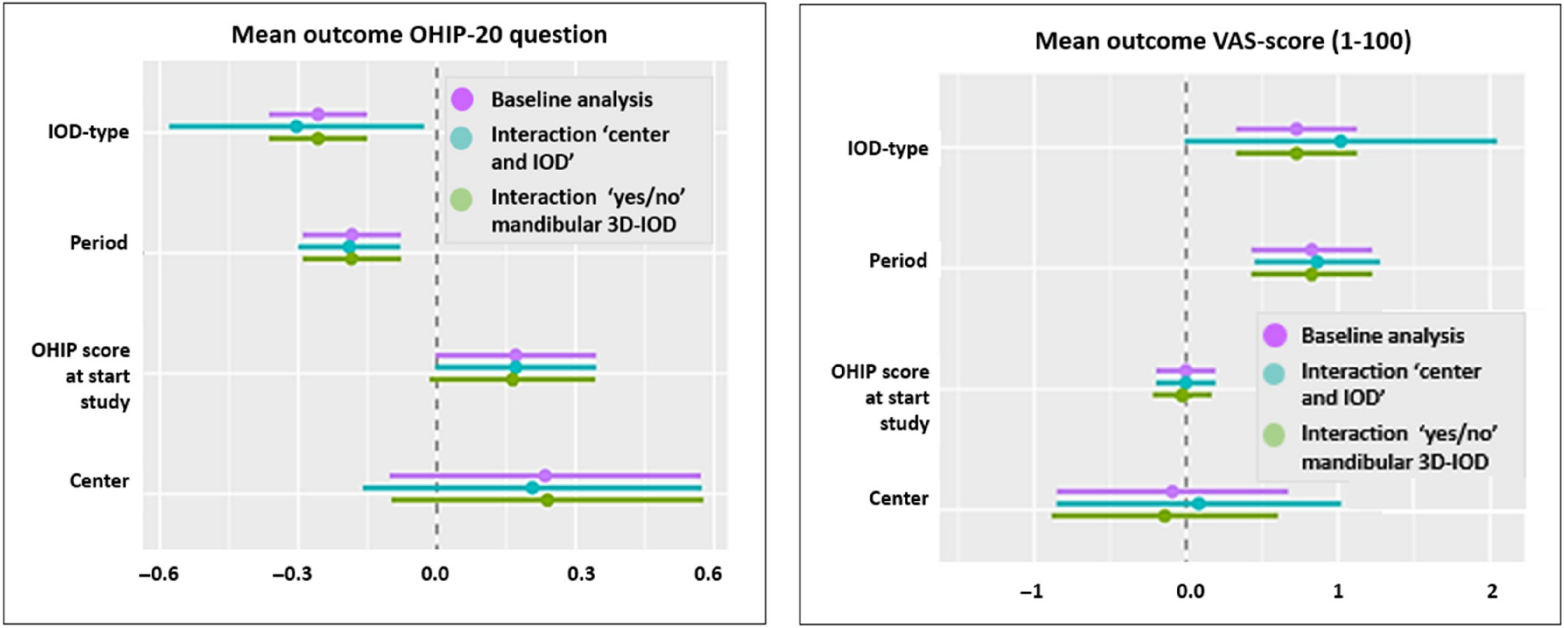
Sensitivity analysis on the OHIP‐20 (left) and VAS score (right): interaction of “center and IOD” and “yes/no” mandibular 3D‐IOD’ (i.e. mandibular 3D‐IOD vs. mandibular C‐IOD); regarding IOD‐type (i.e. C‐IOD vs. 3D‐IOD); Period (i.e. first vs. second year); OHIP score at the start of the study (i.e. OHIP‐20 score at baseline); Center (i.e. treated in Breda vs. Nijmegen, representing maxillary bar vs. maxillary Locators™).

Subsequently, a sensitivity analysis (Figure 5) was performed to assess if the “center” effect was caused by the fact that C‐IODs on conventional bars were provided only in Breda and C‐IODs on Locators™ only in Nijmegen (Figure 5). However, the effect (0.056 for OHIP‐20 and 0.348 for VAS) was small and non‐significant.

Also, no significant effect was observed for wearing a mandibular C‐IOD or a mandibular 3D‐IOD compared with the baseline analysis, showing only a small effect (0.046 for OHIP‐20 and 0.392 for VAS).

With respect to the different domains, the 3D‐IOD scored significantly better for functional limitation (gain 0.586; *p* = 0.000), physical pain (gain 0.315; *p* = 0.001), physical discomfort (gain 0.216; *p* = 0.020), and physical disability (gain‐0.272; *p* = 0.001) than the C‐IOD (Table 3).

**TABLE 3:** The 3D‐IOD versus the C‐IOD; differences for the various OHIP‐20 domains (scores are presented as mean OHIP‐20 points (0–4), as well as for the SF‐36 (0–100).

OHIP‐20 domains				
	Estimate	*p*‐value	2.5%	97.5%
Functional limitation	−0.586	<0.000*	−0.878	−0.295
Physical pain	−0.315	<0.001*	−0.486	−0.144
Psychological discomfort	−0.216	0.020*	−0.388	−0.044
Physical disability	−0.272	<0.001*	−0.421	−0.123
Psychological disability	−0.080	0.117	−0.177	0.017
Social disability	−0.040	0.465	−0.146	0.066
Handicap	−0.143	0.135	−0.326	0.039

SF‐36 domains				
	Estimate	*p*‐value	2.5%	97.5%
Physical functioning	0.078	0.969	−3.824	3.981
Limited due to physical health	−8.873	0.282	−24.703	6.958
Limited due to emotional problems	10.327	0.152	−3.269	23.922
Energy	3.995	0.110	−0.752	8.719
Emotional well‐being	4.504	0.033*	0.568	8.423
Social functioning	4.191	0.193	−1.966	10.348
Pain	0.172	0.968	−8.160	8.503
General health	0.093	0.971	−4.871	5.057

* *p* ≤ 0.05.

#### 4.1.1 VAS (PS)

At the baseline (CD), and after wearing the C‐IOD or 3D‐IOD for 1 year, the VAS outcomes were 25.6, 79.8, and 87.6 points, respectively (Figure 5). As such, the 3D‐IOD scored 7.8 points higher than the C‐IOD in PS (Table 2). The MRA (Table 2) showed a significant effect for “IOD type” and “period,” but not for “OHIP score at baseline” (Table 2).

#### 4.1.2 SF‐36 questionnaire (QoL)

The 3D‐IOD was only significantly preferred in the context of “emotional well‐being” (4.5% higher; *p* = 0.033) (Table 3).

## 5 DISCUSSION

### 5.1 Patients

Before this study, it was expected that the transition from one IOD type to the other could be complicated by peri‐implant mucosal swelling; however, this was not the case. Furthermore, patients did not object to the switch from C‐IOD to 3D‐IOD, or vice versa. To verify whether the study was really blinded for the participants, all were asked at the end of the study if they were confident about the type of IOD they were wearing at that moment; 14 patients guessed correctly and 18 guessed incorrectly. Of the latter group, two patients were convinced they wore a 3D‐IOD when in fact a C‐IOD on Locators™ was installed. Apparently, patients were more concerned about the aesthetics and functionality of their IOD, rather than whether it was handmade or digitally manufactured. At the end of the study, patients were invited to decide which IOD type should be finally placed; 11 patients preferred the C‐IOD and 21 preferred the 3D‐IOD.

### 5.2 Implants

Participants in this study were recruited from edentulous patients referred to the hospital departments for oral maxillofacial surgery in Nijmegen and Breda for maxillary bone augmentation.

By providing implants with a roughened surface, their success rate (SR) has greatly increased in enlarged edentulous maxilla, becoming almost as high as in pristine maxillary bone., varying between 91% and 100%^42^; this was also corroborated in the present study (SR: 97.2%).

### 5.3 Prosthetics

#### 5.3.1 Maxillary IOD

The decision to choose for a bar‐retained IOD, or an IOD on single abutments depends on the personal favor and experience of the dentist. In Nijmegen, as standard procedure for the upper jaw, the Locator™ system was chosen. In Breda, the preferred approach was the use of a titanium bar.

Generally, when 4–6 implants have been installed in the maxillary arch, a bar attachment is propagated,^17^, ^43^ because stresses are more equally distributed and cross‐arch stabilization is induced.^44^ Also, the SR is higher for the splinted implants (bar attachment) than for individual, thus unsplinted, implants.^17^, ^44^ Moreover, bar attachments have the advantage of managing non‐parallelism using angulated abutments.^45^, ^46^

Locators™ are freestanding and can be ideally used in cases of reduced height. Due to the male attachment/patrix being made from nylon, micromovements are allowed. Furthermore, for Locators™ improved peri‐implant hygiene parameters were scored.^47^

In a randomized controlled trial, patients (*n* = 50) wearing a maxillary IOD retained by either Locators™ or a bar were compared. After 5 years, with respect to chewing ability and PROMS (OHIP‐49), no differences were observed for the two attachment types.^48^ This was corroborated in our study as the sensitivity analysis showed that no differences in OHIP‐20 and PS‐score were present due to the difference between an attachment and Locator™ attachment.

#### 5.3.2 Mandibular IOD

Both in Breda and Nijmegen a bar attachment was preferred in the lower jaw. For edentulous mandibles, a recent meta‐analysis stressed that a bar attachment provides the most superior retention. An individual attachment system may be a favorable choice only in case of limited interarch space and parallel implant placement.

At the start of this study in 2017, PEEK had already been introduced as a promising material. It can be easily CAD/CAM milled and its use as a sliding mechanism on bars was first suggested by Spies et al.^49^ Subsequently, others used PEEK as individual inserts in housings in a maxillary overdenture.^50^ In mandibular IODs, as compared with conventional metal housings, PEEK housings showed higher PS regarding retention, stability, and speech,^51^ together with a significantly lower incidence of female housing wear and clip fracture/renewal. In our study, the retention of the PEEK sliding mechanism was satisfactory for maxillary IODs, as demonstrated by the favorable OHIP‐20 and PS outcome.

Regarding a mandibular C‐IOD using bar/metal clip attachment and a 3D‐IOD using a bar/PEEK attachment, the sensitivity analysis pointed out that no significant difference was seen. Between both modalities, only a small difference (0.046 OHIP‐20 point) was calculated.

### 5.4 Non‐inferiority analysis—MRA analysis

The mean improvement of 0.257 points (see “IOD type” in Figure 4) per OHIP‐20 question for the 3D‐IOD (*p* = 0.001), was unexpected as our aim was to only show non‐inferiority.

There is a tendency that each “OHIP point of disapproval at baseline” also implied a more negative judgment of the final IOD (mean effect: 0.171; *p* = 0.069; Table 2), irrespective of IOD type, indicating that the more that patients are disappointed about their CD, the more they are dissatisfied with their final IOD. A negative judgment of both the CD and the final IOD may point to a negative emotional state, caused by adverse emotions, such as feelings of guilt, envy, anger, anxiety, and depressed mood, which may lead to a lower OHRQoL,^52^ particularly because psychosocial impact is one of the four dimensions of OHRQoL.^53^, ^54^ In advance of the treatment, these items should therefore be discussed with the patient. Because these lower OHRQoL scores were reported for both IODs, this did not affect the non‐inferiority analysis.

A relatively high “center” effect of 0.234 points was presented (see “Center” in Table 2), with Nijmegen scoring worse than Breda; however, this was not significant (*p* = 0.191), because of due to the relatively high CI. The latter is explained by the study design, which aimed to examine the effect of “IOD type,” not “center.”

The subsequent sensitivity analysis (Figure 5) denies that the “center” effect was caused by the fact that C‐IODs on conventional bars were provided only in Breda and C‐IODs on Locators™ only in Nijmegen (Figure 5). A possible explanation of the “center” effect is the population variety: in Breda, patients were more inclined to be friendly to the researcher and more grateful to participate in this study.

As it was judged that the effects of the dentures on PROMs would be limited to the period using that very denture, carry‐over effects were not expected; therefore, no washout period was incorporated in the study design.^55^

In the second period of the study (Table 2), patients scored on average 0.184 points lower, thus better, than in the first period, regardless of which type of IOD was worn at that time (*p* = 0.002). This contrasts with our expectation that, after a negative experience with a CD, the first intervention would tend to be scored more positively than the second intervention. An explanation could be that patients had to adjust to the phenomenon of an implant‐borne construction during the transition from the CD to the first IOD, while patients were already used to it during the transition from the first to the second IOD.

Both groups were the same size, so any period effects would be expected to have been canceled out.^55^

### 5.5 PROMs

This study was designed according to the latest International Team for Implantology consensus on reporting PROMs in implant dentistry.^56^ Although other questionnaires are recognized, the OHIP‐20 is especially effective and well‐focused for use with edentulous patients.^57^

An important fact is that all patients were dissatisfied with their upper CD. Previous studies with patients who were pleased with their maxillary CD, reported almost no improvement in general PS, stability, retention, aesthetics, mastication, or speech when IODs were installed.^13^, ^58^ The effects of implants on OHRQoL are greater in patients who requested implants themselves.^59^ In our study, patients complained about the loss of retention of their upper CD. All were highly motivated, as shown by their willingness to undergo an augmentation procedure beforehand. All expressed high expectations about the outcome, which may explain the improvement in OHIP‐20 points for both the C‐IOD and the 3D‐IOD.

To allow for a comparison between various OHIP scales in other publications, the total score was converted to a scale of 0–1, as was propagated in a recent review about mandibular IODs.^60^

For direct normalization: $\mathrm{xn}=\frac{x-\min}{\max-\min}$ was used, for inverted normalization $\mathrm{xn}=\frac{\max-x}{\max-\min}$, where *xn* is the normalized value, *x* is the value in the original tool scale, and *max* and *min* are the highest and lowest values of the original tool scale, respectively.

Tables 4 and 5 list randomized clinical trials (RCTs) related to maxillary and mandibular IODs, applying the OHIP method to measure the OHRQoL. In Table 4, the opposing dentition is specified, while in Table 5, representing mandibular IODs, an upper CD was present in all patients.

**TABLE 4:** Studies for maxillary IODs are shown, in which all OHIP scores were converted from their original scale to values ranging between 0 and 1.

Author	Type of upper prosthesis	Opposing mandibular dentition	Type of OHIP	Effect (0–1)
Aboelez et al., 2022^61^	IOD‐4 on ball	IOD‐2	OHIP‐14 (1–56)	0.89
	IOD‐4 on Locator	IOD‐2		0.98
Al‐Zubeidi et al., 2012^62^	CD	IOD‐2	OHIP‐14 (1–56)	0.77
	IOD‐3‐splinted	IOD‐2		0.90
	IOD‐3‐unsplinted			0.90
Anadioti et al., 2019^63^	IOD‐4 on Locator	IOD (41%), natural dentition (48%), FIP (11%)	OHIP‐49 (0–196)	0.88
Bouhy et al., 2020^64^	CD	natural dentition	OHIP‐20 (20–120)	0.71
	IOD‐4 on Locator	natural dentition		0.91
Martínez‐González et al., 2013^65^	IOD‐4	IOD‐2	OHIP‐19 (0–76)	0.78
	IFP‐8	IFP‐6 (45%) or natural dentition 55%)		0.80
Mo et al., 2022^66^	CD	natural dentition (55%), IFP (13%) IOD (32%)	OHIP‐20 (0–80)	0.26
	IOD‐3 on Locator	natural dentition (55%), IFP (13%), IOD (32%)		0.69
Van Doorne et al., 2021^67^	CD	Natural dentition	OHIP‐14 (0–56)	0.62
	IOD‐6 on MDI	Natural dentition		0.88
Onclin, et al. 2023^68^	IOD‐2 on Locator	IOD‐2	OHIP‐49 (0–196)	0.92
	IOD‐4‐ on Locator	IOD‐2		0.91
Van de Winkel et al., 2024 (this study)	CD	IOD‐2	OHIP‐20 (0–80)	0.43
	C‐IOD‐6	C‐IOD‐2		0.85
	3D‐IOD‐6	3D‐C‐IOD‐2		0.92

Abbreviations: IFP‐8, implant fixed prosthesis on eight implants; IOD‐2, IOD on two implants; IOD‐4, IOD on four implants; IOD‐6, IOD on six implants; MDI, mini dental implant.

**TABLE 5:** Studies for mandibular IODs are shown, in which all OHIP scores were converted from their original scale to values ranging between 0 and 1.

Author	Lower IOD opposed to maxillary CDs	Type of OHIP	Effect (0–1)
Alfadda & Attard, 2017^69^	CD “old” (baseline)	OHIP‐20 (0–80)	0.11
	CD new		0.36
	IOD‐2 on bar		0.65
Attard et al., 2006^70^	CD “old” (baseline)	OHIP‐20 (20–100)	0.36
	CD new		0.63
	IOD‐2 on bar		0.95
Della Vecchia et al., 2018^71^	CD “old” (baseline)	OHIP‐EDENT (0–38)	0.58
	IOD‐2		0.84
	IOD‐2 on MDIs		0.92
	IOD‐4 on MDI		0.95
Heydecke et al., 2005^72^	CD “old” (baseline)	OHIP‐20 (0–80)	0.3
	CD new		0.41
	IOD‐2 on ball		0.61
Jawad et al., 2017^73^	IOD‐2 on ball	OHIP‐20 (20–120)	0.64
	IOD‐2 on MDI		0.79
Matthys et al., 2019^74^	CD “old”	OHIP‐14 (0–56)	0.64
	IOD‐2		0.95

*Note*: An upper CD was present in all patients.

Although crossover studies are more intensive, they are worth the effort because they reduce the sd, reaching statistical significance sooner with fewer patients. Only three crossover studies used the OHIP methodology for edentulous maxillae. The first two studies compared maxillary IODs “with and without” palatal coverage,^75^, ^76^ while the other compared ball retention and Locators™.^61^

As shown in Tables 4 and 5, it is important to realize that the calculated OHIP values (0–1) should be interpreted with care and within the context of the performed studies. This is illustrated, for example, by the fact that the OHIP value (0.95) for a mandibular IOD‐2 compared with a maxillary CD^70^ showed a similar value (0.89–0.98) as for a mandibular IOD‐2 versus a maxillary IOD‐4.^61^

Most studies only show whether one treatment is more successful than the other, but scored values are difficult to compare between studies. OHRQoL measurements in patients are, therefore, always relative, and often set against a baseline in an RCT. In a crossover study, more pertinent conclusions can be drawn; indeed, in our study, a 3D‐IOD functioned better than a C‐IOD, yielding a high + score (Table 4).

#### 5.5.1 SF‐36 questionnaire

Hardly any significant differences were detected between the two IOD types on any of the SF‐36 subscales, suggesting that the oral health status is largely independent from general health status. A statistical change was only found between the C‐IOD and 3D‐IOD in the context of “well‐being,” which was greater in the latter.

In earlier reports, none of the SF‐36 scale scores was statistically different, for example between subjects wearing CDs and dentate controls,^77^ or patients wearing CDs or IODs.^13^ The SF‐36 domains are not very sensitive to changes in oral health as it includes questions on a wide range of health aspects, such as the ability to climb stairs, to walk a mile, or to be full of energy, which are hardly influenced by changes in oral condition. Although statistically significant, more “well‐being” was detected in patients wearing a 3D‐IOD.

### 5.6 Why was the 3D‐IOD preferred over the C‐IOD?

The most logical explanation is that the retention of the PEEK attachment was more than sufficient. Furthermore, due to the strength of the CAD/CAM milled PMMA, the 3D‐IOD could be made slender and therefore can be compared with a removable full arch bridge. Also, the palate remained more uncovered by the 3D‐IOD than the C‐IOD (see Figures 1 and 2), although both IOD types can be defined as free of palatal coverage. Surprisingly, the discussion about palatal coverage is still ongoing. In one study, in which patients wore a maxillary IOD supported by two implants with palatal coverage for 2 months and then wore the IOD without palatal coverage for another 2 months, or vice versa, no differences in OHIP points were calculated.^76^ In another crossover study, using maxillary IODs on four implants, no difference in PS was observed with or without palatal coverage^75^; by contrast, Al‐Zubeidi et al. reported that 85% of the patients preferred no palatal coverage.^62^

A limitation of this study was that, due to unknown implant brands, it was not possible to place a 3D‐IOD in the lower jaw of 10 patients. Furthermore, no uniformity was present between the attachment systems of the maxillary C‐IODs: in Nijmegen, all 27 patients wore a C‐IOD supported by Locators™, whereas in Breda the other five participants were provided with a bar‐supported C‐IOD.

The present study demonstrated that a fully digital workflow, including registration of the maxillomandibular relationship, can produce permanent IODs milled from prefabricated PMMA discs, using PEEK as the sliding mechanism and titanium as a bar. Whether digital techniques also reduce the number of treatment sessions and costs, as has been claimed,^78^ remains to be thoroughly investigated.

## 6 CONCLUSION

It may be concluded that digitally designed and CAD‐CAM–produced supported overdentures (3D‐IODs) performed better than C‐IODs with respect to the OHRQoL. Their slender design, together with the well‐functioning PEEK attachment system, may explain the high PS score and the low, thus improved, OHIP‐20 score for the 3D‐IODs when compared with C‐IODs.

## AUTHOR CONTRIBUTIONS

Thomas Van de Winkel conceptualized the project idea, conducted the investigation, and drafted the manuscript. Frans Delfos designed the digital workflow. Olleke van der Heijden advised on the clinical design of the study. Ewald Bronkhorst performed the statistics. Luc Verhamme and Gert Meijer critically reviewed and revised the manuscript.

## FUNDING INFORMATION

This study was funded by the governmental organization Zorg Onderzoek Nederland Medische Wetenschappen ZonMw under grant 843002808.

## CONFLICT OF INTEREST STATEMENT

The authors declare no conflicts of interest.

## ETHICS STATEMENT

The present study was conducted in accordance with the Declaration of Helsinki and approved by the Ethics Committee of Arnhem/Nijmegen, NL 2017‐3671, December 12, 2017 (Dossier number: 2017‐3671 NL‐number: NL63073.091.17).

## Data Availability

The data that support the findings of this study are openly available in DANS at https://doi.org/10.17026/dans-25s-6cdk, reference number 10.17026/dans‐25s‐6cdk.
